# Pneumococcal lower respiratory tract infections in adults: an observational case-control study in primary care in Belgium

**DOI:** 10.1186/s12875-015-0282-1

**Published:** 2015-05-27

**Authors:** Johan Flamaing, Wilfried De Backer, Yves Van Laethem, Stéphane Heijmans, Annick Mignon

**Affiliations:** Department of Geriatric Medicine, University Hospitals of Leuven, Herestraat 49, B-3000 Leuven, Belgium; Department of Clinical and Experimental Medicine, KU Leuven, Herestraat 49, B-3000 Leuven, Belgium; Department of Pulmonary Medicine, University Hospital and University of Antwerp, 10 Wilrijkstraat, B-2650 Edegem, Belgium; Department of Infectiology, University Hospital Saint-Pierre, 322 Rue Haute, B-1000 Brussels, Belgium; Clinical Research Network, Researchlink, 78 Stationstraat, B-1630 Linkebeek, Belgium; Medical Affairs, Pfizer Vaccines, 17 Boulevard de la Plaine, B-1050 Brussels, Belgium

**Keywords:** Lower respiratory tract infection (LRTI), Primary care, General practitioner, *Streptococcus pneumoniae*, Pneumonia, Belgium, BinaxNOW assay, Serotype-specific urine antigen detection assay

## Abstract

**Background:**

Serious lower respiratory tract infections (SLRTIs), especially *Streptococcus pneumonia*e (SP)-related pneumonia cause considerable morbidity and mortality. Chest imaging, sputum and blood culture are not routinely obtained by general practitioners (GPs). Antibiotic therapy is usually started empirically. The BinaxNOW® and Urine Antigen Detection (UAD) assays have been developed respectively to detect a common antigen from all pneumococcal strains and the 13 pneumococcal serotypes present in the vaccine Prevenar 13® (PCV13).

**Methods:**

OPUS-B was a multicentre, prospective, case-control, observational study of patients with SLRTI in primary care in Belgium, conducted during two winter seasons (2011–2013). A urine sample was collected at baseline for the urine assays. GPs were blinded to the results. All patients with a positive BinaxNOW® test and twice as much randomly selected BinaxNOW® negative patients were followed up. Recorded data included: socio-demographics, medical history, vaccination history, clinical symptoms, CRB-65 score, treatments, hospitalization, blood cultures, healthcare use, EQ-5D score. The objectives were to evaluate the percentage of SP SLRTI within the total number of SLRTIs, to assess the percentage of SP serotypes and to compare the burden of disease between pneumococcal and non-pneumococcal SLRTIs.

**Results:**

There were 26 patients with a BinaxNOW® positive test and 518 patients with a BinaxNOW® negative test. The proportion of pneumococcal SLRTI was 4.8 % (95 % CI: 3.1 %–7.2 %). Sixty-eight percent of positive cases showed serotypes represented in PCV13. In the BinaxNOW-positive patients, women were more numerous, there was less exposure to young children, seasonal influenza vaccination was less frequent, COPD was more frequent, the body temperature and the number of breaths per minute were higher, the systolic blood pressure was lower, the frequency of sputum, infiltrate, chest pain, muscle ache, confusion/disorientation, diarrhoea, pneumonia and exacerbations of COPD was more frequent, EQ-5D index and VAS scale were lower, the number of visits to the GP, of working days lost and of days patients needed assistance were higher.

**Conclusions:**

SP was responsible for approximately 5 % of SLRTIs observed in primary care in Belgium. Pneumococcal infection was associated with a significant increase in morbidity. Sixty-eight percent of serotypes causing SLRTI were potentially preventable by PCV13.

## Background

Lower respiratory tract infections (LRTIs), and more particularly community-acquired pneumonia (CAP), cause considerable morbidity and mortality in adults, especially in the elderly. In Europe, the overall annual incidence of CAP in adults ranges between 1.07 and 1.20 per 1,000 person-years, 14 per 1,000 person-years in adults aged ≥65 years and up to 40 per 1,000 person-years in patients over 80 years [1]. In the USA, pneumonia occurs in about 12 persons per 1,000 annually, and its incidence is highest among persons at the extremes of the age range. It is the sixth leading cause of death in the United States [2].

Clinical symptoms alone do not allow the definite identification of serious LRTI (SLRTI) aetiology. Diagnostic procedures such as chest imaging, sputum and blood culture, recommended by international guidelines [3, 4], are not routinely obtained in primary care and do not allow a timely or definite diagnosis. Antibiotic therapy is usually started empirically and targeted to limit the risk of hospitalization and mortality by pneumococcal pneumonia [2, 5, 6].

Epidemiological surveys demonstrate the importance of *Streptococcus pneumoniae* as the primary pathogen in CAP [7–9]. However, most data on pneumococcal disease originate from invasive disease in hospitalised patients. Data in non-hospitalised or ambulatory patients are very limited. Due to the lack of timely and sensitive diagnostic tools, the burden of pneumococcal disease in SLRTIs is underestimated in general practice [10].

According to the British Thoracic Society guidelines for the management of CAP in adults (update 2009), pneumococcal urine antigen tests should be performed for all patients with moderate or high severity CAP. A rapid testing and reporting service for pneumococcal urine antigen should be available to all hospitals admitting patients with CAP [4]. The BinaxNOW® *S. pneumoniae* is an easy-to-use, urine-based, immunochromatographic membrane test, taking around 15 min to obtain the result, that allows detecting a common antigen from all pneumococcal strains with a sensitivity of 86 % and a specificity of 94 % [11, 12]. A Luminex technology-based multiplex urinary antigen detection (UAD) has been validated to identify 13 pneumococcal serotypes (serotypes 1, 3, 4, 5, 6A/C, 6B, 7 F/A, 9 V/A, 14, 18, 19A, 19 F, 23F, all present in the 13-valent pneumococcal conjugated vaccine [PCV13] Prevenar 13®). UAD has a sensitivity of 97 % and a specificity of 100 % [13]. In the recently published CAPITA trial [14] evaluating the efficacy of PCV13 versus placebo in adults 65 years of age or older, the UAD assay allowed to determine a 45.6 % reduction in vaccine-type CAP.

The proportion of European patients consulting in primary care with LRTI, which receives antibiotics, ranges from 27 % in the Netherlands to 75 % in the United Kingdom [15, 16]. In the large prospective international comparative study GRACE, concerning the management of acute cough among adults in primary care, considerable variation in the 13 countries studied was found. Major differences in the decision whether or not to prescribe an antibiotic in these settings remained, even after adjustment for clinical presentation (symptoms, duration of illness, smoking, age, comorbidity, and temperature). Patients included by a network based in Belgium (Antwerp) were at least two times less likely to be prescribed antibiotics (25 %) than average (53 %) [17]. Prescription of antibiotics is nevertheless very much needed in SLRTIs because of possible serious complications and because of the higher efficiency of antibiotics when prescribed earlier in the course of the disease.

OPUS-B was a multicentre prospective observational (non-interventional) study of patients with SLRTI in primary care in Belgium, conducted during two winter seasons (2011–2013). This study is one of the first studies that combined BinaxNOW® *S. pneumoniae* and UAD assays for identification of pneumococcal infection in urine samples. Its primary objectives were to evaluate the percentage of *S. pneumoniae* SLRTI within the total number of SLRTIs and to evaluate the percentage of serotype specific *S. pneumoniae* SLRTI using the serotype specific UAD. Its secondary objectives were to compare the burden of disease in terms of healthcare use and loss of functionality between pneumococcal SLRTI and non-pneumococcal SLRTI, to describe common practices and antibiotic use and to estimate the proportion of pneumococcal SLRTI that could be prevented by vaccination with PCV13.

## Methods

The OPUS-B study was a multicentre, prospective, case-control, observational and epidemiological trial in primary care. The objective was to enrol 670 patients (see sample size analysis below) with a SLRTI over two winter seasons (from end February 2011 till end March 2013).

The study was conducted in accordance with Good Clinical Practice and the Declaration of Helsinki. An informed consent was obtained from all participants and the study protocol was approved by the Ethics Committee of the University Hospital of Leuven. The presence of a SLRTI was defined by the following criteria: new or worsening cough AND shortness of breath or wheezing or chest pain or new auscultation abnormalities AND rapid variation of temperature: >38.5 °C for patients ≤60 years; >38 °C or <36 °C for patients >60 years AND general signs including malaise, asthenia, headache, myalgia, perspiring/sweating, shivers or confusion AND absence of sore throat and rhinorrhoea. Participation in another clinical trial and prior inclusion into this study were the two exclusion criteria.

A urine sample was collected at the baseline visit. This sample was sent to a central laboratory (Institut de Biologie Clinique, Université Libre de Bruxelles, Brussels, Belgium) for the BinaxNOW assay and for preparation, storage and shipping to Pfizer Vaccines Research and Early Development lab, Pearl River, PA, USA, to perform the UAD assays. General practitioners (GPs) were blinded to the BinaxNOW results to avoid any influence of the test on the patient’s further treatment.

All patients with a positive BinaxNOW test and approximately twice as much randomly selected BinaxNOW negative (case-control) patients had a follow up by the GP.

The physician was entirely free to undertake any imaging investigations (X-rays) and to treat the patient according to his/her own routine practice.

The following data were recorded in all patients using an electronic case report form: socio-demographics, medical history, vaccination history, clinical symptoms, CRB-65 score (Confusion, Respiratory rate, Blood pressure and Age ≥65 years; score 0 [best] to 4 [worst]) [18, 19], LRTI management including pharmacological and non-pharmacological treatments, hospitalization, complementary investigations (including chest X-rays), blood cultures, healthcare use, productivity assessment and functional status (EQ-5D index score) (EuroQol, Rotterdam, The Netherlands, http://www.euroqol.org/; the index score being determined according to Agency for Healthcare Research and Quality [20]; the lowest the score the worse the patient’s health status) including its visual analogue scale (VAS; 0 = worst health you can imagine and 100 = best health you can imagine). Clinical signs, LRTI management, functional status, additional investigations and productivity assessment were recorded at each re-consultation up to 4 to 5 weeks after inclusion in the selected BinaxNOW-positive and -negative patients.

The sample size was not defined on the basis of a pre-defined hypothesis testing. Taking into account a midpoint prevalence of pneumococcal CAP of 8.5 % among all cases of adult SLRTI managed by GPs in the community [21], the total number of patients to include into the study to achieve 50 BinaxNOW-positive cases would be 588 patients. The sensitivity of the BinaxNOW assay has been shown to be 86 % meaning that the percentage of false negative was going to be around 14 %. Therefore, it was estimated that the number of patients to include into the study was to be around 670 patients.

IBM-SPSS Statistics (Version 21.0) was used for the statistical analyses. Missing data were not replaced nor extrapolated. Classical descriptive statistics were used throughout the analyses. Main frequencies were accompanied by 95 % Clopper-Pearson’s confidence intervals (CIs). Patients with a pneumococcal SLRTI were compared to patients with a non-pneumococcal SLRTI. Continuous variables were compared between the two groups of patients using Mann-Whitney’s tests. Discrete variables were compared between the two groups using chi square tests, Fisher’s exact tests or Mann-Whitney’s tests, as appropriate. Taking into account the multiplicity of comparisons (around 65), a Bonferroni’s correction was made on the *p* value which was divided by 65. Therefore a *p*-value <0.00075 was considered statistically significant. *P* value between 0.00075 and 0.049 were considered indicative only of a potential difference between the two groups.

## Results

**Fig. 1:**
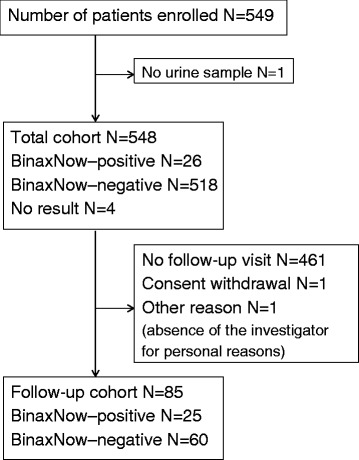
Study enrollment

Overall, 549 patients were enrolled into the study by 38 GPs between February 2011 and March 2013. No urine sample was available for one patient. The total cohort therefore included 548 patients (26 patients with a BinaxNOW positive test, 518 patients with a BinaxNOW negative test and 4 patients for whom the laboratory result was not available). In the follow-up cohort, there were 85 patients: 25 patients with a BinaxNOW positive test (1 patient was lost to follow-up after the baseline assessment) and 60 patients with a BinaxNOW negative test (Fig. 1).

Demographics, medical history, clinical symptoms, diagnostic tests, treatments and functional status of the patients can be found in Table 1. Briefly, the 548 patients (254 males [46.4 %] and 294 females [53.6 %]) were 54.8 ± 18.1 years old (18 to 93 years). An important proportion of patients were retired (36.1 %). Half of the patients (52 %) had regular contacts with young children. Current smokers represented 31.2 % of patients. The vast majority of patients lived at home (96.7 %). An influenza vaccine had been administered to 28.5 % of patients and the 23-pneumococcal polysaccharide vaccine (23-PPV) to 11.7 % of patients. Overall, 53.1 % patients were suffering from chronic diseases. An allergy to antibiotics was reported by 7.7 % of patients. The CRB-65 score was equal to 0 (best health status) and 3 (worst health status) in 55.8 % and 2.4 % of patients, respectively. X-ray radiography was performed in 9.1 % of patients. Pleural effusion was noted in 4.0 % of patients. A blood culture was performed in 3 patients. It was positive in only 1 patient and *Streptococcus pneumoniae* was identified. The tentative diagnosis was bronchopneumonia and pneumonia in 18.1 % and 13.9 % of patients. A pharmacological treatment was prescribed in the vast majority of patients (98.5 %). The antibiotic treatment lasted for a mean of 8.3 ± 1.9 days (3 to 19 days). A non-pharmacological treatment was used in 6.4 % of patients. Sick leave lasted a mean of 5.6 ± 2.7 days (1 to 21 days). Supplementary home care was needed in 2.7 % of patients and 1.5 % patients were referred to the hospital. The mean EQ-5D score was 0.61 ± 0.29. The mean value reported for the VAS scale was 51.7 ± 17.5 mm (midpoint between worst and best possible health status).

**Table 1:** Demographics, medical history, clinical symptoms, diagnostic tests, treatments and functional status of primary care patients with a serious lower respiratory tract infection (N = 548; Total cohort)

	Categories	Mean ± SD or N (%)
Demographics		
Age (year)		54.8 ± 18.1
Age group	18–49 years	208 (38.0)
	50–64 years	165 (30.1)
	≥65 years	175 (31.9)
Gender	Male	254 (46.4)
	Female	294 (53.6)
Smoking status	Smoker	171 (31.2)
	Former smoker	120 (21.9)
	Non-smoker	257 (46.9)
Activity and revenue	Without revenue	64 (11.7)
	Replacement revenue	44 (8.0)
	Self-employed	19 (3.5)
	Manual worker	112 (20.4)
	Employee	101 (18.4)
	Manager	10 (1.8)
	Retired	198 (36.1)
Exposition to young children		285 (52.0)
Living conditions	At home	530 (96.7)
	Nursing home	15 (2.7)
	Institution	3 (0.6)
Medical history		
Influenza vaccination	18–49 years	18 (8.6)
	50–64 years	45 (27.3)
	≥65 years	93 (53.1)
Pneumococcal vaccination	18–49 years	6 (2.9)
	50–64 years	10 (6.1)
	≥65 years	48 (27.4)
Co-morbidities	Asthma	64 (22.0)
	Chronic bronchitis	61 (21.0)
	Diabetes	45 (15.5)
	Renal insufficiency	32 (11.0)
	COPD	94 (32.3)
	Cardiovascular illness	96 (33.0)
	Immunological illness	15 (5.2)
Allergy to antibiotics		42 (7.7)
Clinical symptoms		
Body temperature taken by the physician (°C)		38.3 ± 0.9
Body temperature taken by the patient (°C)		38.9 ± 0.5
Breaths per minute		21.7 ± 7.3
Systolic blood pressure (mmHg)		125.1 ± 17.1
Diastolic blood pressure (mmHg)		76.7 ± 9.2
Cough		537 (98.0)
Sputum		428 (78.1)
Shortness of breath		362 (66.1)
Wheezing		239 (43.6)
Coryza		34 (6.2)
Breath sounds		443 (80.8)
Chest pain		310 (56.6)
Muscle ache		338 (61.7)
Headache		303 (55.3)
Disturbed sleep		303 (55.3)
Feeling unwell		459 (83.8)
Shivers		258 (47.1)
Interference with normal activity		374 (68.2)
Confusion/disorientation		48 (8.8)
Diarrhoea		66 (12.0)
CRB-65 score	0 (best health status)	306 (55.8)
	1	163 (29.7)
	2	66 (12.0)
	3 (worst health status)	13 (2.4)
Diagnostic tests		
X-ray radiography		50 (9.1)
X-ray signs	No signs	15 (30.0)
	Lobar consolidation	9 (18.0)
	Infiltrate	26 (52.0)
Pleural effusion		2 (4.0)
Blood culture		3 (0.5)
Tentative diagnosis	Acute bronchitis	292 (53.3)
	Bronchopneumonia	99 (18.1)
	Pneumonia	76 (13.9)
	COPD exacerbation	81 (14.8)
Treatment		
Pharmacological treatment		540 (98.5)
	Antibiotics	490 (90.7)
	Antipyretics	425 (78.7)
	NSAIDs	24 (4.4)
	Corticosteroids	91 (16.9)
	Bronchodilators	135 (25.0)
	Antitussives	259 (48.0)
	Low weight heparin	6 (1.1)
	N-acetylcysteine	102 (18.9)
Non-pharmacological treatment		35 (6.4)
	Oxygen	9 (25.7)
	Physiotherapy	30 (85.7)
Sick leave		234 (42.7)
Supplementary home care		15 (2.7)
Hospitalisation		8 (1.5)
Functional status		
EQ-5D index		0.61 ± 0.29
EQ-5D visual analogue scale (mm)		51.7 ± 17.5

SD = Standard Deviation; COPD = Chronic Obstructive Pulmonary Disease; °C = Celsius degree; mmHg = millimetre of mercury; CRB-65 = Confusion, Respiratory rate, Blood Pressure and Age ≥65 years [16, 17]; NSAID = Non-Steroidal Anti-Inflammatory Drug; EQ-5D index (http://www.euroqol.org/ and reference [18])

Among the 85 patients participating in a complete follow-up visit: 76.5 % had recovered, 18.8 % had improved with residual symptoms, 1.2 % had no improvement and 3.6 % had worsened symptoms. The mean number of visits to the GPs was 1.62 ± 1.41. The number of work days lost was 5.3 ± 7.1. The number of days patients needed external assistance was 3.5 ± 7.7.

Comparisons between non-pneumococcal (BinaxNOW-negative) and pneumococcal (BinaxNOW-positive) SLRTIs can be found in Table 2. In the BinaxNOW-positive patients, women were more numerous (p = 0.016), there was less exposure to young children (p = 0.009), seasonal influenza vaccination was less frequent (p = 0.037), COPD was more frequent (p = 0.002), the body temperature measured by the physician (p < 0.0001) and the number of breaths per minute (p = 0.001) were higher, the systolic blood pressure was lower (p < 0.0001), sputum was produced more frequently (p = 0.026), the frequency of chest pain (p = 0.004), muscle ache (p = 0.040), confusion/disorientation (p = 0.001) and diarrhoea (p < 0.0001) was higher, infiltrate was more frequent (p = 0.030), pneumonia, bronchopneumonia and infectious exacerbations of COPD were more frequent (p < 0.0001), non-pharmacological treatments (physiotherapy) were more frequent (p = 0.018), EQ-5D index (p < 0.0001) and VAS scale (p = 0.039) were lower, translating a worse health status. All the other differences between the two groups were not statistically significant (p > 0.050).

**Table 2:** Comparison of BinaxNOW-positive (pneumococcal) and -negative (non-pneumococcal) patients with serious lower respiratory tract infection (Total and Follow-Up cohorts)

	Categories	Non-Pneumococcal SLRTI (N = 518)	Pneumococcal SLRTI (N = 26)	*P* value
		Mean ± SD or N (%)	Mean ± SD or N (%)	
Total cohort				
Demographics				
Age		55.1 ± 18.1	52.2 ± 19.2	>0.050
Age group	18-49 years	195 (37.6)	11 (42.3)	>0.050
	50-64 years	157 (30.4)	6 (23.1)	
	≥65 years	166 (32.0)	9 (34.6)	
Gender	Male	246 (47.5)	6 (23.1)	0.016
	Female	272 (52.5)	20 (76.9)	
Smoking status	Smoker	160 (30.9)	9 (34.6)	>0.050
	Former smoker	115 (22.2)	4 (15.4)	
	Non-smoker	243 (46.9)	13 (50.0	
Activity and revenue	Without revenue	57 (11.0)	5 (19.2)	>0.050
	Replacement revenue	40 (7.7)	4 (15.4)	
	Self-employed	18 (3.5)	1 (3.8)	
	Manual worker	106 (20.5)	6 (23.1)	
	Employee	99 (19.1)	1 (3.8)	
	Manager	9 (1.7)	0 (0.0)	
	Retired	189 (36.5)	9 (34.6)	
Exposition to young children		277 (53.6)	7 (26.9)	0.009
Living conditions	At home	500 (96.5)	26 (100.0)	>0.050
	Nursing home	15 (2.9)	0 (0.0)	
	Institution	3 (0.6)	0 (0.0)	
Medical history				
Influenza vaccination		141 (27.2)	2 (7.7)	0.037
Pneumococcal vaccination		60 (11.6)	4 (15.4)	>0.050
Co-morbidities	Asthma	57 (11.0)	6 (23.1)	>0.050
	Chronic bronchitis	55 (10.6)	6 (23.1)	>0.050
	Diabetes	42 (8.1)	3 (11.5)	>0.050
	Renal insufficiency	28 (5.4)	4 (15.4)	>0.050
	COPD	83 (16.0)	11 (42.3)	0.002
	Cardiovascular illness	92 (17.8)	4 (15.4)	>0.050
	Immunological illness	6 (1.2)	1 (3.8)	>0.050
Allergy to antibiotics		41 (7.9)	1 (3.8)	>0.050
Clinical symptoms				
Body temperature taken by the physician (°C)		38.2 ± 0.9	38.9 ± 0.6	<0.0001
Body temperature taken by the patient (°C)		38.9 ± 0.5	39.0 ± 0.1	>0.050
Breaths per minute		21.5 ± 7.2	27.1 ± 6.2	0.001
Systolic blood pressure (mmHg)		128 ± 57	111 ± 18	<0.0001
Diastolic blood pressure		77 ± 9.2	74 ± 9.1	>0.050
Cough		507 (97.9)	26 (100.0)	>0.050
Sputum		399 (77.0)	25 (96.2)	0.026
Shortness of breath		341 (65.8)	19 (73.1)	>0.050
Wheezing		227 (43.8)	10 (38.5)	>0.050
Coryza		34 (6.6)	0 (0.0)	>0.050
Breath sounds		417 (80.5)	22 (84.6)	>0.050
Chest pain		286 (55.2)	22 (84.6)	0.004
Muscle ache		314 (60.6)	21 (80.8)	0.040
Headache		282 (54.4)	18 (69.2)	>0.050
Disturbed sleep		284 (54.8)	17 (65.4)	>0.050
Feeling unwell		433 (83.6)	23 (88.5)	>0.050
Shivers		241 (46.5)	16 (61.5)	>0.050
Interference with normal activity		351 (67.8)	21 (80.8)	>0.050
Confusion/disorientation		40 (7.7)	8 (30.8)	0.001
Diarrhoea		55 (10.6)	11 (42.3)	<0.0001
CRB-65 score	0 (best)	292 (56.4)	11 (42.3)	>0.050
	1	156 (30.1)	6 (23.1)	
	2	61 (11.8)	5 (19.2)	
	3 (worst)	9 (1.7)	4 (15.4)	
Diagnostic tests				
X-ray radiography		44 (8.5)	5 (19.2)	>0.050
X-ray signs	No signs	9 (1.7)	0 (0.0)	>0.050
	Lobar consolidation	7 (1.4)	2 (7.7)	>0.050
	Infiltrate	22 (4.2)	4 (15.4)	0.030
Pleural effusion		2 (7.4)	0 (0.0)	>0.050
Blood culture		2 (0.4)	1 (3.8)	>0.050
Tentative diagnosis	Acute bronchitis	288 (55.6)	1 (3.8)	<0.0001
	Bronchopneumonia	87 (16.8)	11 (42.3)	
	Pneumonia	70 (13.5)	6 (23.1)	
	COPD exacerbation	73 (14.1)	8 (30.8)	
Treatment				
Pharmacological treatment		511 (98.6)	25 (96.2)	>0.050
	Antibiotics	462 (89.2)	24 (92.3)	>0.050
	Antipyretics	405 (78.2)	18 (69.2)	>0.050
	NSAIDs	23 (4.4)	1 (3.8)	>0.050
	Corticosteroids	83 (16.0)	7 (26.9)	>0.050
	Bronchodilators	130 (25.1)	5 (19.2)	>0.050
	Antitussives	240 (46.3)	17 (65.4)	>0.050
	Low weight heparin	5 (1.0)	1 (3.8)	>0.050
	N-acetylcysteine	98 (18.9)	2 (7.7)	>0.050
Non-pharmacological treatment		29 (5.6)	5 (19.2)	0.018
	Oxygen	8 (1.5)	1 (3.8)	>0.050
	Physiotherapy	25 (4.8)	5 (19.2)	0.010
Sick leave		220 (42.5)	11 (42.3)	>0.050
Supplementary home care		14 (2.7)	1 (3.8)	>0.050
Hospitalisation		6 (1.2)	2 (7.7)	>0.050
Functional status				
EQ-5D index		0.62 ± 0.28	0.35 ± 0.35	<0.0001
EQ-5D visual analogue scale (mm)		52 ± 17	44 ± 22	0.039
Follow-up cohort				
Visits to GPs		1.27 ± 1.30	2.44 ± 1.36	0.001
Working days lost		3.49 ± 4.83	9.56 ± 9.46	<0.0001
Number of days patients needed assistance		2.14 ± 6.34	6.64 ± 9.70	0.014

SD = Standard Deviation; COPD = Chronic Obstructive Pulmonary Disease; °C = Celsius degree; mmHg = millimetre of mercury; CRB-65 = Confusion, Respiratory rate, Blood Pressure and Age ≥65 years [16, 17]; NSAID = Non-Steroidal Anti-Inflammatory Drug; EQ-5D index (http://www.euroqol.org/ and reference [18]); GP = General Practitioner; *P* value = statistical probability of Mann-Whitney, Chi-square or Fisher’s exact tests as appropriate. Statistical significance for *p* values <0.00075 (Bonferroni’s correction for the multiplicity of analyses)

In the follow-up cohort, the number of visits to the GP (p = 0.001), the number of working days lost (p < 0.0001) and the number of days patients needed assistance (p = 0.014) were higher in the BinaxNOW-positive patients (Table 2).

In the total cohort, the proportion of pneumococcal SLRTI was equal to 4.8 % (26 out of 544 cases; 95 % CI: 3.2 %–6.9 %).

The cross-table with the results of the BinaxNOW and UAD assays can be found in Table 3. Eighty-eight potentially contaminated samples had to be eliminated from the UAD assay. There were no differences at baseline between the patients who had their sample eliminated and those who were actually analysed (data not shown). Taking into account the positive samples from both BinaxNOW and UAD assays, the proportion of pneumococcal SLRTI among the SLRTI patients was equal to 4.8 % (22 out of 456 cases; 95 % CI: 3.1 %–7.2 %). Fifteen (15) of these 22 positive cases (68.2 %) showed pneumococcal serotypes represented in the PCV13. The number and proportions of the different serotypes among the 15 UAD positive samples can be found in Table 4.

**Table 3:** Cross-table of the results of the BinaxNOW and Urine Antigen Detection assays*

		BinaxNOW assay		Total N(%)
		Negative N(%)	Positive N(%)	
Urine Antigen Detection Assay	Negative N(%)	434 (95.2)	7 (1.5)	441 (96.7)
	Positive N(%)	10 (2.2)	5 (1.1)	15 (3.3)
	Total N (%)	444 (97.4)	12 (2.6)	456 (100.0)

*: 88 contaminated samples were eliminated from the analysis

**Table 4:** Number and proportion of the serotypes in pneumococcal serious lower respiratory tract infections using the Urine Antigen Detection (UAD) assay

Pneumococcal serotype	N	%
1	1	6.67
3	1	6.67
5	1	6.67
6A	2	13.33
7 F	2	13.33
14	2	13.33
18C	1	6.67
19A	5	33.33
Total*	15	100.0

*15 (3.3 %) out of 456 SLRTI cases were positive for the UAD assay

## Discussion

This study contributes to the knowledge of pneumococcal LRTIs in primary care in Europe and particularly in Belgium. Indeed, most data are generated on pneumococcal invasive disease in hospital settings, where culture methods to identify the pathogens are available. This is also one of the rare studies to combine BinaxNOW® *S. pneumoniae* and UAD assays for the identification of pneumococcal antigens in urine samples. It confirmed, in agreement with previous studies [10, 13], that rapid urine antigen assays (in clinical routine practice the BinaxNOW test would take around 15 min to obtain the results) can determine SLRTI aetiology in adults and quickly identify a group of patients in whom antibiotic treatment should be considered.

The proportion (4.8 %) of pneumococcal infections among primary care patients diagnosed with a SLRTI was globally in agreement with the previous epidemiological studies [21, 22].

The burden of SLRTIs and particularly of pneumococcal SLRTIs has been highlighted in many other studies [7, 8, 21, 23] and is confirmed by the current study. The proportion of European patients consulting in primary care with LRTI, which receives antibiotics, ranges from 27 % in the Netherlands to 75 % in the United Kingdom [15, 16]. In our study, the percentage of antibiotic prescription was around 90 %. This could be due to the fact that our patients presented with SLRTIs, were quite old (55 years in average) and appeared to have severe comorbidities: 32 % had a COPD, 33 % had a cardiovascular disease and 15.5 % had diabetes.

The comorbidity profile of our patients was in agreement with the one published by Torres et al. [1]: 9.4–62 % COPD, 3–50 % asthma, 10–47.2 % chronic heart disease, 4.9–33 % diabetes and 0.5–26.7 % chronic renal diseases. In the study of Ryan et al. [24], elderly patients with COPD had nearly six-times the incidence of pneumonia compared with those without COPD.

The CRB-65 score was the worst (level 2 or 3) in 34.6 % of patients with a pneumococcal versus 13.5 % of patients with a non-pneumococcal SLRTI. Although not statistically significant, this difference is important since a higher CRB-65 score also correlates with pneumonia severity and the risk of death [19].

Social-economic deprivation is considered as a risk factor for invasive pneumococcal disease [25] and for CAP [26]. The same tendency was observed in our study, 34.6 % of patients with a pneumococcal disease being without revenue or with replacement revenue. Although not statistically significantly different, this was almost twice the percentage of patients without a pneumococcal disease (18.7 %). All patients are not equal when facing SLRTIs and economic deprivation is a parameter that should be taken into account when considering the prescription of antibiotics and vaccination coverage.

In terms of clinical symptoms potentially differentiating a bacterial from a viral LRTI, fever, headache, the absence of diarrhoea and the presence of an infiltrate at the X-ray radiography predict a bacterial aetiology [5]. This was confirmed in our study for the presence of an infiltrate at the X-ray radiography but not for diarrhoea. The tentative diagnosis of pneumonia was more frequent in pneumococcal versus non-pneumococcal LRTIs both in our study (65 % versus 30 %) and in the study of Holm et al. [7] (33 % versus 17 %).

The CAPiTA trial has demonstrated 45.6 % efficacy of PCV13 against vaccine-type pneumococcal pneumonia, 45.0 % efficacy against vaccine-type non-bacteremic pneumococcal pneumonia and 75.0 % efficacy against vaccine-type invasive pneumococcal disease among adults aged ≥65 years [14, 27]. In our study, 68 % of serotypes causing pneumococcal SLRTI were potentially preventable by the PCV13. On August 13, 2014, the ACIP recommended routine use of PCV13 among adults aged ≥65 years. PCV13 should be administered in series with the 23-PPV, the vaccine currently recommended for adults aged ≥65 years [27].

The limitations of the current study are intrinsic to its observational, epidemiological and real-life design. All general practitioners of the study could freely prescribe the examinations, laboratory analyses and treatments, they considered clinically relevant and practical. In clinical primary care in Belgium, GPs do not routinely refer patients for a chest X-ray in case of SLRTI. Moreover, the results of radiography are sometimes available only after a few days. This explains the low uptake of chest radiography (50 patients [9.1 %]). The collection of a urine sample for the BinaxNOW was the only intervention required by the protocol. To avoid any influence of the result of the test on patient’s follow-up, all urine analyses were performed by a central laboratory and the physicians remained blinded to its results in the setting of our study. The investigators were not able to enrol 670 patients and the observed frequency of pneumococcal infection being significantly lower than the forecasted 8.5 %, it was not possible to reach 50 BinaxNOW-positive patients. Eighty-eight samples were contaminated and were therefore discarded from the UAD assay. Limitations are also inherent to the sensitivity and specificity of the assays. Non-PCV13 serotypes are not identified by the UAD assay. The multiplicity of statistical inferential tests was taken into account. *P* values lower than 0.00075 have to be considered statistically significant but higher values should be considered indicative only and should be interpreted cautiously. Moreover, the results should not be extrapolated to other populations or other time periods without caution.

## Conclusions

In this study, *Streptococcus pneumoniae* was responsible for approximately 5 % of SLRTIs observed in primary care in Belgium. Pneumococcal infections were associated with clinically and statistically significant increases of morbidity. Sixty-eight percent (68 %) of serotypes causing SLRTI were potentially preventable by the PCV13.

## Abbreviations

CAP: Community-acquired pneumonia
CI: Confidence interval
CRB-65: Confusion, Respiratory rate, Blood pressure, Age ≥65 years
GP: General practitioner
LRTI: Lower Respiratory Tract Infection
PCV13: Pneumococcal Conjugated Vaccine 13 serotypes (Prevenar 13®)
SLRTI: Serious Lower Respiratory Tract Infection
UAD: Urine antigen detection
VAS: Visual analogue scale
