# Erratum to: Pneumococcal lower respiratory tract infections in adults: an observational case–control study in primary care in Belgium

**DOI:** 10.1186/s12875-016-0420-4

**Published:** 2016-03-02

**Authors:** Johan Flamaing, Wilfried De Backer, Yves Van Laethem, Stéphane Heijmans, Annick Mignon

**Affiliations:** Department of Geriatric Medicine, University Hospitals of Leuven, Herestraat 49, B-3000 Leuven, Belgium; Department of Clinical and Experimental Medicine, KU Leuven, Herestraat 49, B-3000 Leuven, Belgium; Department of Pulmonary Medicine, University Hospital and University of Antwerp, 10 Wilrijkstraat, B-2650 Edegem, Belgium; Department of Infectiology, University Hospital Saint-Pierre, 322 Rue Haute, B-1000 Brussels, Belgium; Clinical Research Network, Researchlink, 78 Stationstraat, B-1630 Linkebeek, Belgium; Medical Affairs, Pfizer Vaccines, 17 Boulevard de la Plaine, B-1050 Brussels, Belgium

Due to a change in reagent affecting the performance of the Urinary Antigen Detection (UAD) assay, the positivity cut-off values of the assay had to be revised. A number of epidemiological studies have been affected by this change, including the original version of this article [1].

All samples of the study have been reanalysed and the impact on the study results can be found in the attached Excel sheet (Additional file 1):

- Due to change in cut-off for serotype 5 and 14, there is one case of serotype 5 and of serotype 14 less.
- The revision of the analysis showed 1 additional positive results for serotype 18C and for serotype 23 F
- Overall the total number of positive UAD results has not changed.

Results of the assay are shown in the corrected Tables 3 and 4, included in this erratum.

**Table 3:** Cross-table of the results of the BinaxNOW and Urine Antigen Detection assays^a^

		BinaxNOW assay		Total N(%)
		Negative N(%)	Positive N(%)	
Urine Antigen Detection Assay	Negative N (%)	433 (95.0)	8 (1.7)	441 (96.7)
	Positive N (%)	11 (2.4)	4 (0.9)	15 (3.3)
Total N (%)		444 (97.4)	12 (2.6)	456 (100.0)

^a^88 contaminated samples were eliminated from the analysis

**Table 4:** Number and proportion of the serotypes in pneumococcal serious lower respiratory tract infections using the Urine Antigen Detection (UAD) assay

Pneumococcal serotype	N	%
1	1	6.67
3	1	6.67
6A	2	13.33
7 F	2	13.33
14	1	6.67
18C	2	13.33
19A	5	33.33
23 F	1	6.67
Total^a^	15	100.0

^a^15 (3.3 %) out of 456 SLRTI cases were positive for the UAD assay

## Additional file

Additional file 1:
**Changes in UAD results.** (XLSX 12 kb)
